# Editorial: Machine learning for cybersecurity

**DOI:** 10.3389/frai.2025.1640609

**Published:** 2025-09-30

**Authors:** Chathurika S. Wickramasinghe Brahmana, Daniel Marino, Daswin De Silva, Milos Manic

**Affiliations:** ^1^Capital One, McLean, VA, United States; ^2^Amazon.com Inc., San Diego, CA, United States; ^3^La Trobe University, Melbourne, VIC, Australia; ^4^Virginia Commonwealth University, Richmond, VA, United States

**Keywords:** cybersecurity, Cyber Physical Systems, intelligent systems, explainable AI, artificial intelligence, neural networks

In an era where digital transformation is reshaping every aspect of our lives, Cyber-Physical Systems (CPSs) have emerged as integral components of modern critical infrastructure (Verma et al., 2025; Paul et al., 2025). These systems—embedded in power grids, healthcare services, and transportation networks—leverage computer and information technologies (CITs) to streamline operations, improve productivity, and enable seamless communication within and beyond system boundaries. However, as CPSs grow increasingly interconnected, their exposure to cyber threats has escalated, posing significant risks to economic stability and human safety (Saheed and Misra, 2025; Torba and Jahankhani, 2025).

The growing reliance on Information and Communication Technologies (ICTs) introduces various vulnerabilities, making systems susceptible to data stream manipulation attacks—such as interception, tampering, or deletion—as well as AI-specific threats, including model hijacking, jailbreaking, and data poisoning. These vulnerabilities are particularly troubling in critical infrastructure settings, such as power grids, transportation systems, or healthcare networks, where even minor disruptions can lead to severe consequences (Obasi and Benson, 2025). For example, a cyberattack on a smart grid could interrupt electricity supply to thousands of homes or hospitals, impacting emergency services and risking lives (Sun et al., 2018). Similarly, interference with autonomous vehicle systems could result in collisions, endangering passengers and pedestrians (Ganin et al., 2019). The Microsoft Digital Defense Report 2024 revealed that over 600 million cybercriminal and nation-state attacks are detected daily, spanning threats such as ransomware, phishing, and identity-based attacks (Microsoft Digital Defense Report, 2024). The most targeted sectors include Information Technology (24%), Education and Research (21%), and Government (12%).

Such scenarios underscore how deeply integrated ICTs are in modern critical infrastructure, and how their compromise can cascade into broader societal harm. In many cases, CPSs not only perform essential operations but also rely on real-time data to make automated decisions. This reliance makes them particularly vulnerable to data integrity attacks, where false or manipulated inputs can lead to incorrect and potentially dangerous actions—like administering incorrect medication in hospitals, redirecting traffic into dangerous routes, or causing costly system downtimes and ransom payments (Wells, 2024; Gates, 2024). Moreover, as CPSs become more connected through the Internet of Things (IoT), the attack surface expands exponentially (Xu et al., 2018). Each connected device represents a potential entry point for attackers, who may exploit unpatched software, weak authentication, or insecure communication protocols. Given the scale and critical nature of these systems, a breach can have far-reaching implications not just for the individual or organization affected, but for public safety, economic stability, and national security.

In this context, ensuring the cybersecurity of CPSs has become not just a technical challenge, but a societal imperative (Kayan et al., 2022). It requires a multi-disciplinary approach involving industrial engineering, computer science, systems design, AI, AI ethics, policy-making, and end-user literacy and awareness. Cybersecurity must be designed into these systems from the ground up, not bolted on as an afterthought. Without proactive efforts to secure CPSs, society risks becoming increasingly vulnerable to cyber threats that could disrupt essential services, erode public trust, and ultimately threaten lives.

This special Research Topic brings into focus the transformative potential of Machine Learning (ML) and Artificial Intelligence (AI) in enhancing the cybersecurity of Cyber-Physical Systems (CPSs). As CPSs continue to proliferate across sectors—from smart manufacturing and autonomous vehicles to healthcare and critical infrastructure—they generate vast volumes of heterogeneous and often high-velocity data (Wickramasinghe et al., 2021). This influx of data presents both a challenge and an opportunity: while it can overwhelm traditional security approaches that rely on static rules and manual oversight, it also creates fertile ground for ML and AI algorithms to learn from complex patterns, detect subtle anomalies, and predict potential threats before they materialize.

AI is proving to be a powerful tool in cybersecurity, especially for identifying threats hidden within vast datasets. Unlike traditional detection systems, AI can uncover subtle patterns and anomalies. Supervised learning models, trained on labeled attack data, are effective in identifying known threats. Meanwhile, unsupervised techniques like clustering and dimensionality reduction can detect unusual system behavior that may indicate previously unknown attacks. By recognizing deviations from normal activity, these models enhance early warning capabilities and help defend against emerging cyber threats, making ML a crucial asset in the evolving landscape of digital security (Mavikumbure et al., 2022). AI technologies, especially those involving deep learning and reinforcement learning, offer further enhancements by adapting dynamically to evolving threat landscapes. Moreover, the ability of ML/AI systems to operate autonomously and at scale is crucial in CPS environments where decisions often need to be made in milliseconds. In the case of an autonomous vehicle under attack, for instance, a well-trained ML model holds the potential to significantly enhance the security of control systems by identifying malicious signals in real time.

Over recent decades, the effectiveness of AI/ML has been demonstrated in various cybersecurity applications, including intrusion detection systems (IDS), malware classification, phishing detection, and behavioral analytics. These successes point toward a future where AI- and ML-driven security solutions are not merely supportive but integral to the design and operation of CPSs. As such, the integration of ML and AI into CPS cybersecurity is not just an upgrade—it represents a paradigm shift toward intelligent, adaptive, and proactive defense mechanisms. This Research Topic aims to explore and accelerate this shift by gathering innovative research, practical case studies, and interdisciplinary approaches that push the boundaries of what's possible in secure CPS operations.

For this Research Topic, 12 papers were submitted, of which six were accepted. Five of the accepted papers focus on the use of AI to enhance cybersecurity, while one addresses the ethical considerations surrounding AI usage. Collectively, these papers highlight both the critical role of AI in securing Cyber-Physical Systems (CPSs) and the ethical concerns that researchers must be mindful of when developing AI technologies. A high-level summary of the accepted papers is provided below.

## Protecting digital assets using an ontology-based cyber situational awareness system

Cyber situational awareness is essential for the timely detection and mitigation of cybersecurity threats in increasingly complex digital environments. This study (Almoabady et al.) presents a intelligent framework that integrates Isolation Forest and autoencoder algorithms with the Structured Threat Information Expression (STIX) standard and ontology-based knowledge modeling to enhance real-time threat detection and cyber threat intelligence.

The methodology combines the strengths of Isolation Forest for high-dimensional anomaly detection and autoencoders for non-linear feature learning, enabling a dual-layered, adaptive anomaly detection system. Threat intelligence is structured using the STIX framework, promoting consistent and dynamic information representation. Additionally, the development of a cybersecurity ontology allows for semantic correlation and contextualization of threat indicators, enriching feature mapping and improving the overall effectiveness of detection.

Experimental results validate the efficacy of the proposed approach, achieving 95% accuracy, 99% F1 score, and 94.60% recall, surpassing benchmark models. The integration of STIX and ontological reasoning enhances semantic understanding and standardization of threat data, positioning the framework as a scalable and proactive solution for cyber situational awareness.

This research highlights the limitations of existing intelligence and ontology systems in terms of scalability and performance, and addresses the need for domain-specific knowledge representation to support informed risk management. Future work will focus on real-world deployment, optimization, and extension to broader threat scenarios, contributing to the development of adaptive and resilient cybersecurity ecosystems.

## Explainable correlation-based anomaly detection for industrial control systems

Anomaly detection is critical for ensuring the safety and reliability of Industrial Control Systems (ICS), yet current approaches often overlook the intricate temporal and parameter correlations among devices. This study (Birihanu and Lendák) proposes a novel, explainable correlation-based anomaly detection framework designed specifically for ICS environments. By combining Long Short-Term Memory Autoencoders (LSTM-AE) to determine optimal data window sizes with Pearson correlation analysis, a Latent Correlation Matrix (LCM) is constructed to capture meaningful parameter relationships. A Latent Correlation Vector (LCV) derived from the LCM is then modeled using a Multivariate Gaussian Distribution (MGD) to detect anomalies via a threshold mechanism.

To enhance model transparency and interpretability, the framework integrates Shapley Additive Explanations (SHAP) for both feature selection and root cause analysis. The proposed method is evaluated on benchmark datasets including SWaT, HIL-HAI, and IoT-Modbus, achieving superior performance with up to 96% precision and 84% F1-score. Beyond anomaly detection, the method effectively identifies root causes, assisting ICS engineers and decision-makers in diagnosing system faults.

This resource-efficient approach is tailored for deployment in low-power ICS devices and operates in batch mode for offline analysis. Future work will explore real-time data stream integration, robustness evaluation in real-world scenarios, and the incorporation of advanced deep learning models such as TimesNet. The proposed framework represents a significant step toward interpretable, high-performance anomaly detection in critical industrial environments.

## Credibility-based knowledge graph embedding for identifying social brand advocates

Brand advocates—individuals who voluntarily promote brands through positive word-of-mouth—hold significant influence in shaping customer perceptions and driving engagement. However, identifying these advocates accurately within online platforms remains a challenge due to the lack of intelligent systems capable of capturing complex social interactions and credibility cues. This study (Abu-Salih et al.) presents a novel framework that leverages Knowledge Graphs (KGs) and advanced embedding techniques to identify brand advocates with enhanced accuracy and interpretability.

The proposed approach constructs a domain-specific KG by integrating extended ontologies and semantic repositories to represent user interactions, preferences, and brand affinities. To ensure authenticity and trustworthiness, they embed a social credibility analysis module, extending the DSpamOnto ontology to detect and filter potential spammers in social commerce contexts. The KG is then transformed into a low-dimensional vector space using state-of-the-art graph embedding models, enabling effective link prediction, clustering, and visualization of brand advocacy patterns.

Experimental evaluation demonstrates the framework's effectiveness in accurately identifying genuine brand advocates while maintaining high data reliability through social credibility insights. The study contributes a scalable, ontology-driven solution for brand engagement analysis and lays the foundation for future research in domain adaptation and multimedia data integration, such as image and video sentiment analysis, to enrich KGs and expand applicability across diverse domains.

## Ethics and responsible AI deployment

This work (Radanliev et al.) presents a review of current machine learning methodologies, with a critical emphasis on the often-overlooked ethical implications of artificial intelligence (AI) systems. The study introduces a novel, structured ethical deployment framework that integrates privacy-preserving technologies—such as federated learning, differential privacy, and homomorphic encryption—within the context of international ethical standards and regulatory compliance. By bridging the gap between technical innovation and ethical responsibility, this research contributes a multidimensional perspective on responsible AI development.

The paper explores how AI impacts employment, equity, and societal structures, calling for global cooperation to establish ethical and regulatory frameworks that transcend borders. It advocates for “Ethics by Design,” interdisciplinary collaboration, and adaptive regulatory mechanisms as essential strategies for addressing evolving ethical challenges. Additionally, it underscores the importance of empirical research and advanced risk assessment to inform ethical policies and guide AI implementation across industries such as healthcare, finance, and transportation.

The study highlights that privacy-preserving algorithms are crucial in upholding data confidentiality in AI systems. By demonstrating the practical applications of these technologies, this research lays a foundation for ethically grounded AI innovation and provides a forward-looking blueprint for integrating privacy and ethical principles into future AI systems. The study ultimately offers insights for developers, researchers, and policymakers seeking to align AI technologies with societal values and global standards.

## Exploring security threats and solutions techniques for Internet of Things (IoT): from vulnerabilities to vigilance

The rapid expansion of the Internet of Things (IoT) across industries has transformed how devices interact, communicate, and operate. While offering unprecedented convenience and innovation, this proliferation has introduced significant security challenges that threaten the confidentiality, integrity, and availability of IoT systems. This survey (Sahu and Mazumdar) provides a comprehensive overview of the diverse security threats facing IoT ecosystems, including data breaches, unauthorized access, physical tampering, and denial-of-service attacks.

By analyzing vulnerabilities across multiple layers of the IoT architecture—ranging from sensing and networking to middleware, gateways, and applications—the survey underscores the urgent need for robust, multi-layered security measures. The paper also investigates the potential of emerging technologies such as blockchain, machine learning, and edge computing to enhance IoT security by enabling decentralized trust, intelligent threat detection, and local data processing.

Through an extensive review of existing security frameworks, protocols, and best practices, this survey provides a guide to researchers, industry practitioners, and policymakers in strengthening the resilience of IoT deployments. Furthermore, it identifies critical research gaps and future directions necessary to address evolving cyber threats. As IoT continues to integrate deeper into critical infrastructure and consumer environments, this work advocates for a proactive and collaborative approach to IoT security—ensuring a safer, more reliable digital ecosystem.

## Heuristic machine learning approaches for identifying phishing threats across web and email platforms

In the digital age, while advanced technologies have enhanced convenience and connectivity, they have also given rise to sophisticated cyber threats—phishing being among the most pervasive. Phishing attacks aim to deceive users into revealing sensitive information such as passwords, banking credentials, and personal data by impersonating legitimate sources through URLs, emails, and fake websites. This study proposes a comprehensive, heuristic-based machine learning approach for detecting phishing attacks across multiple vectors—namely, URLs, emails, and websites.

The proposed methodology in this work (Jayaprakash et al.) incorporates data pre-processing steps, including cleaning, feature selection, and transformation, followed by the deployment of machine learning models tailored for each phishing medium. For URL-based phishing detection, 56 features were utilized, achieving a detection accuracy of 97.2%. Email phishing detection yielded an accuracy of 97.4%, while website phishing detection, using 48 selected features, achieved the highest accuracy of 98.1%. Comparative analysis with baseline models—Random Forest, Support Vector Machine (SVM), and Naive Bayes—demonstrates the superior performance of the proposed heuristic-based technique across all evaluated categories.

This research contributes a unified, high-performance framework for phishing detection and offers insights into feature relevance and attack patterns across platforms. Future work will explore extending the model's capabilities to detect multimodal phishing attempts involving images, videos, social engineering tactics, and malicious attachments in various messaging services.

## Discussion and directions

Our goal for this Research Topic was to explore advancements in machine learning for cybersecurity of Cyber-Physical Systems. The accepted papers in this Research Topic showcase key applications of machine learning in this area, such as threat detection and anomaly detection. The papers demonstrate how this topic extends across multiple areas, including industrial control systems, IoT, phishing detection, and social platforms. This highlights the impact machine learning is having across multiple industries in order to improve security, safety, and trustworthiness. At the same time, the adoption of machine learning and AI also poses new challenges. The rapid adoption of AI without proper governance frameworks, ethical guidelines, and robust defense strategies can also cause harm, particularly in mission-critical systems where biased or compromised AI decisions could lead to severe consequences. AI and ML suffer from bias that can lead to inaccurate threat detection and misclassification, are difficult to understand and govern effectively, and are vulnerable to sophisticated attacks, including adversarial attacks where input data is manipulated to mislead models. Additionally, many current cybersecurity datasets are outdated and fail to reflect modern AI-driven threats, while the research community lacks systematic comparisons of AI models for specific cybersecurity domains. Data collection also poses significant challenges to privacy. The proliferation of IoT devices allows convenient collection of data to provide increased personalization, but opens the door to new attack vectors and increases the impact of data breaches. To further advance this Research Topic, we emphasize the need for better governance, more realistic datasets, systematic model comparisons, and exploration of state-of-the-art architectures to improve the trustworthiness of cybersecurity solutions powered by machine learning. We look forward to future advancements in this research area.
